# Using LiDAR Data to Measure the 3D Green Biomass of Beijing Urban Forest in China

**DOI:** 10.1371/journal.pone.0075920

**Published:** 2013-10-11

**Authors:** Cheng He, Matteo Convertino, Zhongke Feng, Siyu Zhang

**Affiliations:** 1 Nanjing Forest Police College, Nanjing, China; 2 School of Public Health-Division of Environmental Health Sciences, University of Minnesota Twin-Cities, Minnesota, United States of America; 3 Institute on the Environment, University of Minnesota Twin-Cities, Minnesota, United States of America; 4 Institute of GIS, RS&GPS, College of Forestry, Beijing Forestry University, Beijing, China; University of Florida, United States of America

## Abstract

The purpose of the paper is to find a new approach to measure 3D green biomass of urban forest and to testify its precision. In this study, the 3D green biomass could be acquired on basis of a remote sensing inversion model in which each standing wood was first scanned by Terrestrial Laser Scanner to catch its point cloud data, then the point cloud picture was opened in a digital mapping data acquisition system to get the elevation in an independent coordinate, and at last the individual volume captured was associated with the remote sensing image in SPOT5(System Probatoired'Observation dela Tarre)by means of such tools as SPSS (Statistical Product and Service Solutions), GIS (Geographic Information System), RS (Remote Sensing) and spatial analysis software (FARO SCENE and Geomagic studio11). The results showed that the 3D green biomass of Beijing urban forest was 399.1295 million m^3^, of which coniferous was 28.7871 million m^3^ and broad-leaf was 370.3424 million m^3^. The accuracy of 3D green biomass was over 85%, comparison with the values from 235 field sample data in a typical sampling way. This suggested that the precision done by the 3D forest green biomass based on the image in SPOT5 could meet requirements. This represents an improvement over the conventional method because it not only provides a basis to evalue indices of Beijing urban greenings, but also introduces a new technique to assess 3D green biomass in other cities.

## Introduction

It is hard to estimate the amount of urban green space due to its characteristics of diverse structure and scattered distribution [1], [2]. Therefore, 3D green biomass could be vividly defined as a 3D volume of the stems and leaves of all plants growing in the region [3], which can not only more accurately reflect the proportion of all vegetations in the region than such traditional 2D indicators as forest area and coverage, but also provide some ecological efficiency and green indexes suitable for the ecological assessment of the urban landscape, while playing an important role in planning the city and building the forestry discipline [4], [5] .

In general, two methods can be used for the 3D Green Biomass estimation: the ground survey and the estimating with the remote sensing technology [6]–[9]. Actually, the ground survey is difficult to be done on a large scale even if the value can get a high accuracy because the green biomass can be acquired by the 3D volume measured by each tree's crown width and diameter at the breast height so that the systems need continuous field tests to be improved [10]–[21]. The remote sensing technology has been widely used in vegetation classification, forest fire monitoring and 3D green biomass measuring. Lv et al. [22] calculated the 3D volume by the crown height and width, how the crown width and height on the aerial photo was first measured. Cheng et al. [24] acquired the 3D volume by using the screen tracking vectorization by means of GIS, who first made some field investigations to get the data of leaf area and vegetation coverage, and then combined the data with some high resolution images (IKONOS). Zhou et al. [25] succeeded in making an estimation of the 3D green biomass of Shanghai urban city forest by classifying the species on the aerial photos with high resolution, then simulating the stereo quantity by the plane quantity on the computer. Compared with the traditional ground work, the remote sensing techniques mentioned above have made greater improvements with such less cost as manpower, material and time, leading a fast calculation of 3D green biomass on a large scale. However, although the approaches can mitigate some problems, their precisions are not guaranteed because they are computered by the crown volume based on an appropriate formula suitable for the crown shape. Additionally, there are so timely and limited calculations that it is difficult to be widely promoted. Therefore, it is essential to find a more precise and generalized approach capable of achieving the 3D green biomass by means of remote sensing retrieval method today when the ecological environment is more and more important.

The spatial distribution of leaf area determines resource capture and canopy exchanges with the atmosphere. It is generally tedious and time-consuming to measure the spatial distribution of leaf area, even when 3D digital techniques are employed [21]–[26]. Many tree models, like light models, therefore choose individual canopies as a volume filled with leaf area. Simple shapes like ellipsoids or frustums have been extensively used to model tree shape [26]–[31]. More sophisticated parametric envelopes have been proposed by Cescatti (1997) to extend the range of modeled canopy shapes, and non-parametric envelopes like polygonal envelopes are expected to fit any tree shape [32]. However, all the envelopes showed that different shape models for the same tree may lead to large differences in crown volume [33]–[36]. None of these methods for tree crown volume estimation has been evaluated by comparison with direct measurements. Moreover, neither method accounts for the fractal nature of plants, because only one value of crown volume is computed (i.e., at the observation scale) and changes in crown volume with measurement scale are ignored.

On the other hand, airborne laser scanners can be used to acquire vertical and horizontal forest structure in detail as scanning targets with laser pulses. In particular, such vertical measurements enable the prediction of forest biomass and carbon storage. Furthermore, laser sensors can be used to accurately measure topographical information, the physical properties of a forest and other information. Therefore, ALS(Auto Scanner Laser System)has been recognized as a more efficient and precise instrument than field surveys and optical remote sensing techniques [37]–[43]. Since the early to mid 1980s, several studies using full waveform sensors have been performed for forest inventory, merchantable timber volume estimation [44], and forest canopy characterization [45]–[47]. Recently, several researchers have applied discretely emitted laser pulses for the individual- and stand-level tree height estimation [47]–[53]and height-based timber volume estimates [44]–[46].However, there is currently no effective approach in the methods mentioned above to resolve the problems on how to calculate the canopy volume accurately and quickly, especially for the volume on a large scale.

In this paper, the 3D green biomass of Beijing urban forest was calculated and analyzed based on the remote sensing retrieval model. This approach, in which to obtain the point cloud data of the crown, 30 different trees in size of each species from over 30 common tree species in Beijing urban area(like *arborvitae, cedar, pine, cypress, ginkgo, poplar, sycamore, willow tree, Sophora japonica, Ailanthus altissima, Koelreuteria, ash, maple, cork oak* and other about ten common tree species)were chosen for scanning with laser scanner FARO(FARO develops and markets portable CMMs (coordinate measuring machines) and 3D imaging devices to solve dimensional metrology problems.), was designed to calculate the 3D green biomass of single wood by CASS (CAD AID SURVEY SYSTEM) software(CASS mapping equipment in the South in AutoCAD 2004 to develop a new generation of digital terrain cadastral mapping software)which has been patented in China in 2011 [24]–[28], associated with the remote sensing image in SPOT5, and by means of SPSS, GIS, RS and other spatial analysis tools [46]–[49].

## Materials and Methods

### Ethics Statement

No specific permits were required for the described field studies, since the trees chosen in the study are owned and managed by the state including the sites for our sampling are not privately-owned or protected in any way and specific permission for non-profit research, therefore, is not required. The field studies were not involved in endangered or protected species in this area.

### Data Acquisition

The study site was located in Beijing (39°26′40″N to 41°03′05″N, 115°25′45″E to 117°30′20″E), The SPOT5 remote sensing image data from four views in summer of 2009 in Beijing were selected in this paper, including resolution of 10 m multi-spectral band and 2.5 m panchromatic band. Besides, there is much supporting information, such as Beijing 1∶250,000 administrative map, traffic road map, water maps, maps of forest resources, Worldview remote sensing images of 2008 in Beijing City, the latest Goolge Earth data and so on. The total area of Beijing is 16,800 km^2^, of which mountainous areas occupy about 62% and plains take up the rest. Forestry areas is 104,609,637 m, including 65,891,408 m forestation-suitable, 557,631 m open forest, 30,580,843 m shrub, 2,110,388 m young forest and 5,469,367 m other forest. Geographically, Beijing is a transitional zone for southern and northern plants of China. Influenced by warm-temperate continental monsoon climate, its sub-natural flora generally belongs to warm temperate zone deciduous broad-leaved forest and coniferous forest, but due to serious destruction in early years, currently there are only small area forests with sporadically scattered trees. In some higher mountainous planted forest, the Larix principis-rupprechtii forests were originally distributed as the sub-natural compositions, however, shrub and secondary forests are the most widely distributed zonal vegetations of Beijing, such as betula, populus, quercus.

**Figure 1:**
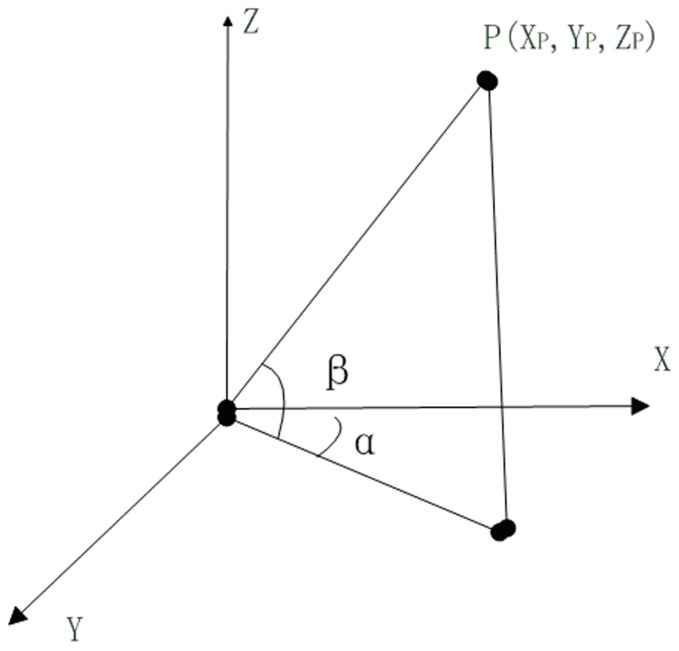
Schematic diagram of scanning point coordinate calculation. 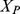
——P abscissa values; 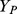
——P ordinate values; 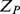
——P Elevation Value; 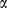
——included angle of P was perpendicular to the YZ plane with X axis; 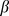
——included angle of P with XY plane.

In this study, at first over 1000 trees (30 species and over 30 trees in each species), like pinus bungeana, cedar and pinus tabulaeformis, planting in campuses, parks, roadsides, housing estates and mountain forests in Beijing city, were sampled and scanned systematically and representatively with terrestrial 3D laser scanner FARO LS880, and then their 3D coordinates were measured by means of some instruments like Trimble GPS(Global Positioning System) and Topcon total station. To acquire the point cloud data, we put up a platform, where some parameters of the scanner should be first set. The horizontal direction was 360°, vertical direction was 155° (from −90 to 65) and a resolution of 2 mm in 10 m. Next, three stations should be set up according to its growth and relative terrain of the tree to be measured, because they can generally constitute an equilateral triangle in theory whose included angle is 120°. Additionally, at least three public spheres should be placed on a non-straight line between two stations and all could be scanned by the above 3 stations without any shelter. The target paper, which only acted as a reference for scanning, were finally posted on the trunk with a height of about 1.3 m above ground northwards (Figure 1). Only when all trees were scanned, could the target ball be removed, otherwise, they had to be re-scanned. To get the volume of the sampling forest, each single tree had to be scanned in about 10 minutes. For field application, scanning for single tree should be done in open forest because too many close planting trees with branches and leaves will lead to serious shadows. Meanwhile, some pictures of the trees to be measured as reference for post-processing point cloud trees should be taken, where the scanner was placed.

**Figure 2:**
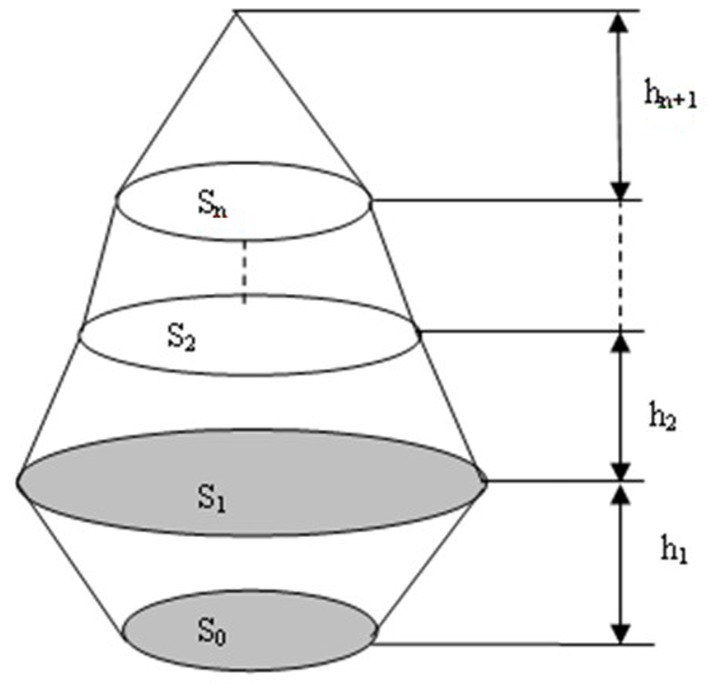
Mimic diagram of three-dimensional laser scanning method for measuring the crown volume.

**Figure 3:**
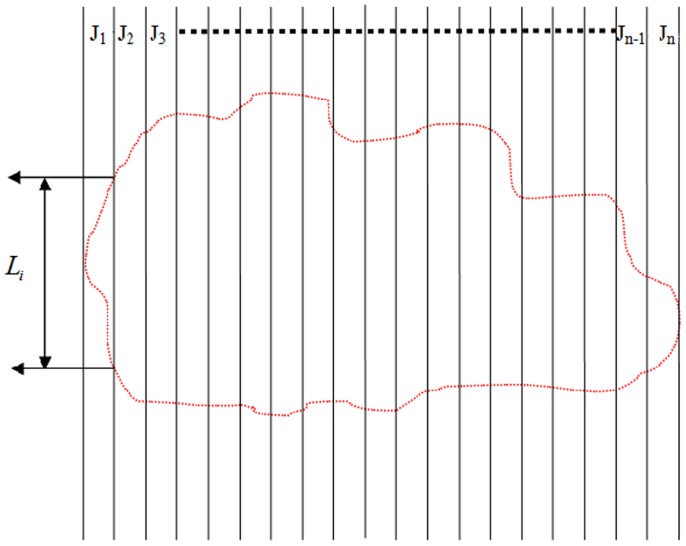
Mimic diagram of measuring crown cross-sectional area.

To achieve the crown 3D green biomass of single tree, we made a calculation on point cloud data of the crown using a CASS software which could computer a volume with digital elevation after a secondary development of CAD (Computer Aided Design). In processing, some pictures with different point clouds of the standing woods were first pieced together at a coordinate system by coordinate match by means of the 3D scanner's software, and then a 3D model of standing wood was developed and saved as .dxf which could be discerned by the digital mapping system after the data were pre-processed and extracted. Next, the point cloud picture captured by the scanner was opened in a digital mapping data acquisition system to set up an independent coordinate system and the elevation could be extracted (Figure 2).The area whose volume should be calculated was outlined with a closed compound line instead of fitting curve in a mesh with 3D triangles, because a fitted curve would be replaced by the broken line so that the precision of the results could be affected. Finally, the point cloud volume was calculated with the help of DTM (Digital Terrain Model) method of the system shown as follow: the points on the crown surface collected at the same height were linked with the smooth curve to form some contour lines which were then separated into some grids with a regular 2 cm cell size(Certainly, the length can be divided into any other size, but 2 cm here was just for convenience), so that the topmost elevation in each grid could be estimated by linear interpolation and marked at its top right, where the designed elevation was set 0; next, we calculated the volumes with some formulas shown as follows:(1) V_cornerpoint_ = h*1/4 S_grid_; (2) V_edgepoint_ = h*2/4S_grid_;(3) V_turningpoint_  = h*3/4 S_grid_;(4) V_midpoint_ = h*S_grid_. (5)V_crown_ = *n*V_grid_, where *h* is a canopy height and *n* is the number of all grids (Figure 2 and 3).

**Figure 4:**
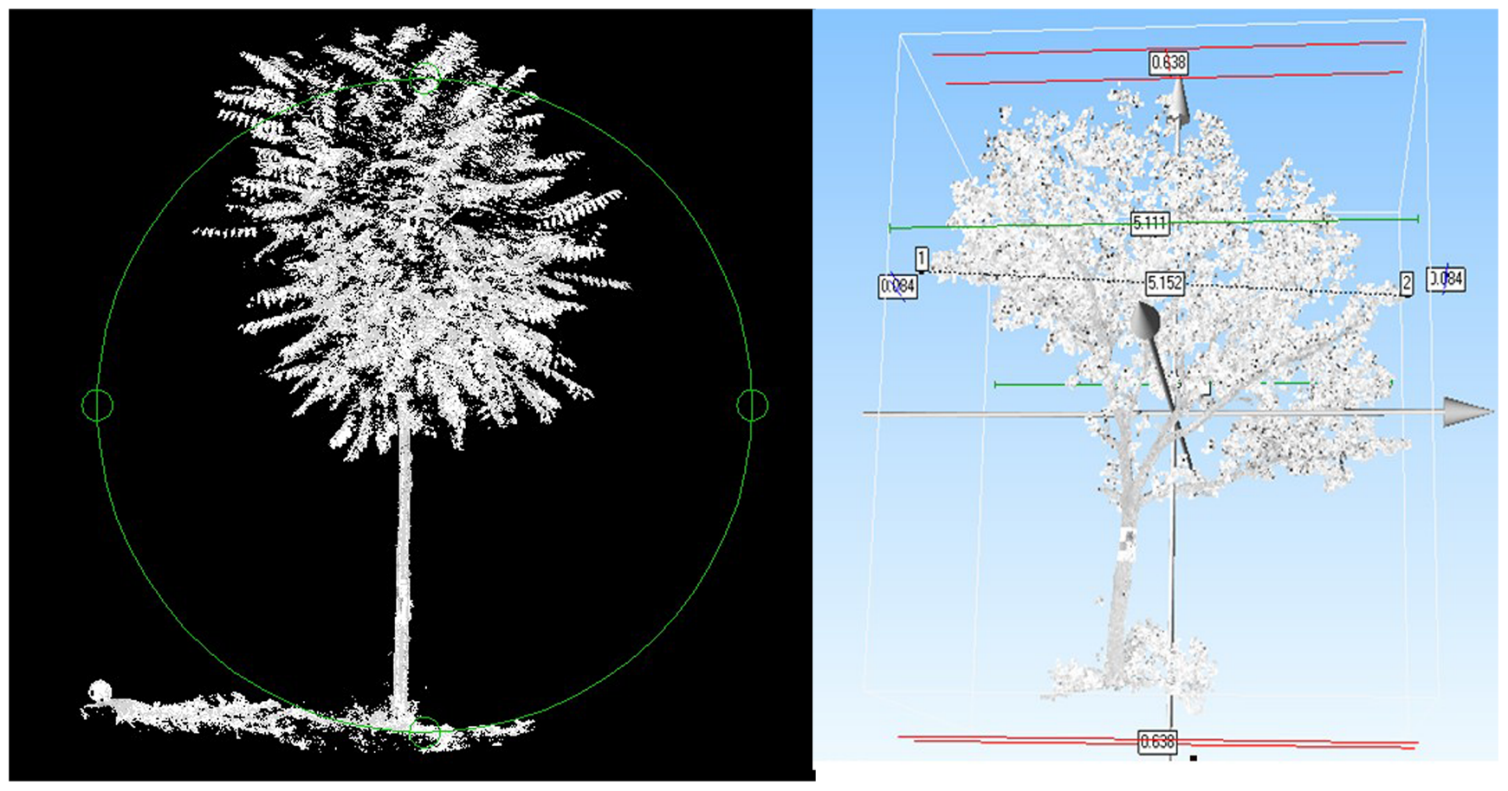
3D point cloud data.

The point cloud data are shown in Figure 4. In remote sensing SPOT5 data, we performed a spatial resolution of 2.5 m panchromatic and 10 m multi-spectral bands using artificial visual interpretation method to extract the vegetation classification information. And we added SPOT5 image gray value, its remote sensing factors and GIS factors into the independent values.

### Remote sensing image classification and information extraction

**Figure 5:**
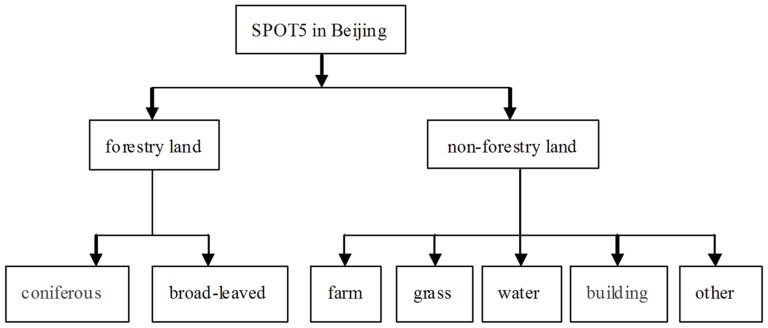
Classification flow diagram of the image information.

**Figure 6:**
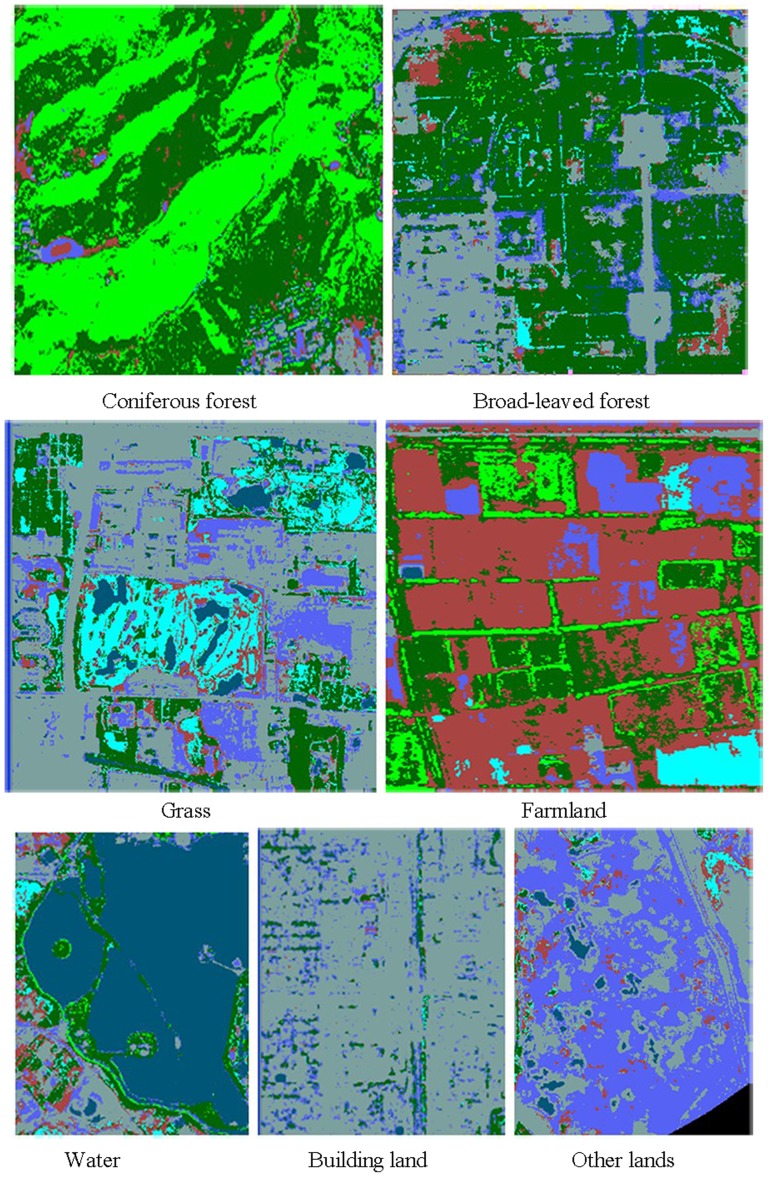
Interpretation signs. Coniferous forest, Broad-leaved forest, Grass, Farmland, Water, Building land and other lands are signed in the figure.

The green biomass retrieval model can be developed by means of various information, methods, monitoring and manual interpretation. In supervised method, at first, some known characteristic parameters are extracted from the spectral features based on samples from training areas, and then other unspecified parameters which can be extracted and classified in sorts from images are analyzed according to prior probability of different kinds of objects. The ground information is demonstrated by pixels in image, while the pixel information is expressed by spectral characteristics of different image bands. Due to the different spatial resolutions and complex grounds, some mixed pixels can appear in images which will result in different objects with the same spectra characteristics or same spectrum with different objects. Therefore, the accuracy will be confined if the urban vegetation is classified only by supervised classification because misclassification and loss classification can arise in some specific classification and extraction. The present vegetation is classified by means of visual interpretation or computer-aided, however, it is of low automation, long hours and low efficiency or likely to be worse because of the unskilled labors or less educated operators (Figure 5 and Figure 6).

In this study, the first information was captured based on the spatial structure feature and spectral brightness of the pixels in different bands of the SPOT5 remote sensing image in Beijing urban regions in 2010. The second was characteristics of landscape, landform and forest resource distribution. The third was about such maps as current vector, forest distribution, greening investigation data, contour and traffic each year.

Beijing is so large that a lot of random points must appear in the accuracy assessment of classification, thereby they should be chosen at absolute random instead of any mandatory rule in practice and the regional classification maps were assessed under the Accuracy Assessment module of ERDAS IMAGINE software(Table 1).

**Table 1:** Table of accuracy totals of classification.

	Reference Totals	Classified Totals	Number Correct	Producer's Accuracy/%	User's Accuracy/%	Kappa
Coniferous forest	14	7	6	42.86	85.71	0.834
Broadleaf forest	15	15	11	73.33	73.33	0.686
Grass	5	5	4	80.00	80.00	0.790
Farmland	11	15	10	90.91	66.67	0.626
Waters	2	2	2	100	100	1
Construction lands	39	39	37	94.87	94.87	0.916
Other lands	14	17	13	92.86	76.47	0.726
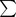	100	100	83			

For classification of remote sensing images in some large areas, its accuracy has been able to meet the needs of the latter analysis and assessment.

### Modeling

The Principal Component Analysis(PCA) with varimax rotation was used for factor analysis in this study. As two PCs can be shown in Table 2, the loading values of each independent variable in either the first PC or the second were little changed, indicating that there was only a small amount of correlation between grouping variables. If the eight variables were forcibly added into the model, it could not be guaranteed to high accuracy that some uncorrelated variables to 3D green biomass could be directly regressed by the model. So the factor involved into modeling could be acquired by automatic filtration with the help of stepwise regression modeling.

**Table 2:** Factors loading rotation matrix of varimax.

Variables	Remote sensing	principal components	
		1	2
1	B1	−0.716	0.696
2	B2	−0.751	0.658
3	B3	−0.741	0.670
4	SWIR	−0.726	0.686
5	NDVI	0.745	0.667
6	SAVI	0.744	0.668
7	MSAVI	0.742	0.670

Note: B1, B2 are visible bands, where B1 can detect absorption and reflectance of plant green hormone, and B2 belongs to the red light zone capable of distinguishing the color of different types of vegetation from the color difference; Where B3 is near-infrared bands, which can reflect the sensitivity of plants to chlorophyll by the correlation between some acquired strong information and factors like leaf area index and biomass; SWIR (short-wave (length) infrared (band)); NDVI (Normalized Difference Vegetation Index); SAVI (Soil-Adjusted Vegetation Index); MSAVI (Modified Soil-Adjusted Vegetation Index).

Based on the results interpreted by the remote sensing images and the green biomass data measured in the plots, the relative remote sensing factors and GIS factors were chosen as the independent values of a model by means of the spatial relationship of RS image rectified by GPS to ground samples, where the remote sensing factors were related to the image gray value in SPOT5 and its linear and nonlinear combination etc, and GIS factors included slope, elevation, aspect etc.(Some factors were not involved in modeling as independent variables like slope and aspect since the subjects in the study were mainly located in Beijing urban areas and topographic relief was not much changed.) The 3D green biomass was repeatedly regressed to model the conifer and broadleaf tree respectively on basis of the correlation between the factors and the green biomass values observed in the plots.

The 3D green biomass model of conifer is shown as follow:
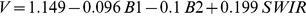
(1)


The 3D green biomass model of broadleaf tree is shown as follow:
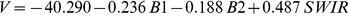
(2)


Where B1, B2 are visible bands, where B1 can detect absorption and reflectance of plant green hormone, and B2 belongs to the red light zone capable of distinguishing the color of different types of vegetation from the color difference; SWIR band which can well reflect the water feature in the plant leaves is a shortwave infrared zone, by which it is easy to make the identification and classification of vegetation, soil, and water.

Based on validation and accuracy assessment on the model and comparison with the actual measured data in the ground, the data for modeling should be systemized, and then 150 samples were added into modeling and 100 checking samples were chosen to test their accuracy.

It is possible to develop a reliable, scientific and operable model. Analysis on the correlation coefficient between the gray values of four bands in SPOT5 and the 3D green biomass of conifer and broadleaf tree by using EXCEL software, the results were shown in Table 3 and 4.

**Table 3:** Correlation coefficient of conifer volume and RS factors.

Variables	Band and combinations	Correlation coefficient with 3D green biomass
1	B1	0.92917
2	B2	0.93602
3	B3	0.93711
4	SWIR	0.93102

**Table 4:** Correlation coefficient of broadleaf tree volume and RS factors.

Variables	Band and combinations	Correlation coefficient with 3D green biomass
1	B1	0.86879
2	B2	0.80498
3	B3	0.805022
4	SWIR	0.87112

All tests were performed using version 18.0 of the SPSS software (SPSS Inc., Chicago, IL).

## Results and Analysis

### Classification results and analysis

All the 3D green biomass of 1015 trees scanned by 3D laser were added in the calibrated remote sensing image so that we could get the gray value at each sampling point, and then by means of remote sensing image Worldview 1 and Google earth with the resolution of 0.5 m, each sampling tree was soon located.

Based on supervised classification and visual interpretation, the total Beijing urban forest area of conifer and broadleaf tree was 275.08 km^2^, of which the area of conifer tree was 54.47 km^2^, while that of broadleaf tree was 220.61 km^2^. The correlation coefficient between the 3D biomass of conifer tree and RS factors was over 0.9 and that of broadleaf tree was over 0.8, thus revealing that the linear correlation was very close [44]–[47], thereby a model of 3D green biomass could be developed with the direct remote sensing data by the multiple linear regression.

### Model checking

After sampling and drying every organ of the tree, we converted them and got the biomass. Meanwhile,the stem and volume were accurately measured by means of sectional measurement (Table 1), As can be seen from Table 1, the overall classification accuracy reaches more than 80%, and Kappa factor of 0.780, exceeding the requirements of 0.7. After inducing and analyzing the eight variable factors of B1, B2, B3, SWIR(short-wave (length) infrared (band)), NDVI (Normalized Difference Vegetation Index), SAVI (Soil-Adjusted Vegetation Index) and RVI (Ratio Vegetation Index), we got the regression equations of conifer and broadleaf tree shown as Eq.1 and Eq.2. The accuracy of the model was tested with the correlation coefficient and F test to evaluate (Table 5).

**Table 5:** Checking table of model precision.

Species	Correlation Coefficient	Square of Correlation Coefficient	Revised Square	Estimation Error	Statistical Analysis			
Conifer tree	0.951	0.904	0.902	2.21	F value	Degree of Freedom1	Degree of Freedom2	Significant Change
					411.651	3	147	0.0000
Broadleaf tree	0.894	0.799	0.792	7.67	F value	Degree of Freedom1	Degree of Freedom2	Significant Change
					622.611	3	478	0.0000

**Figure 7:**
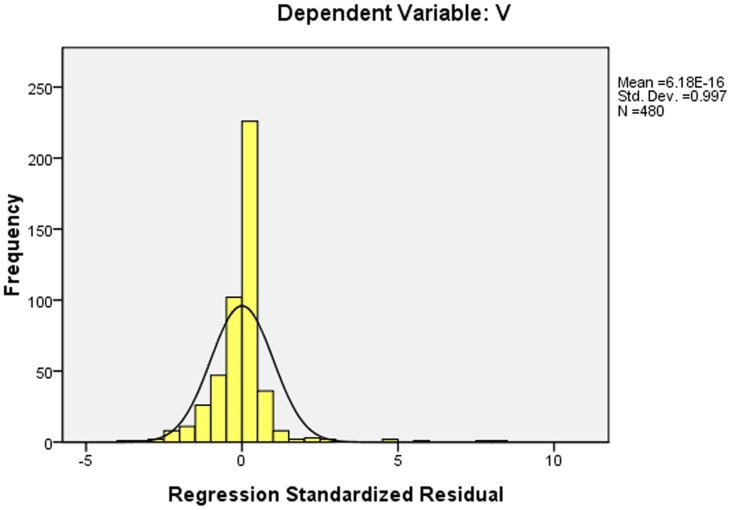
Histogram of regression standardized residual.

**Figure 8:**
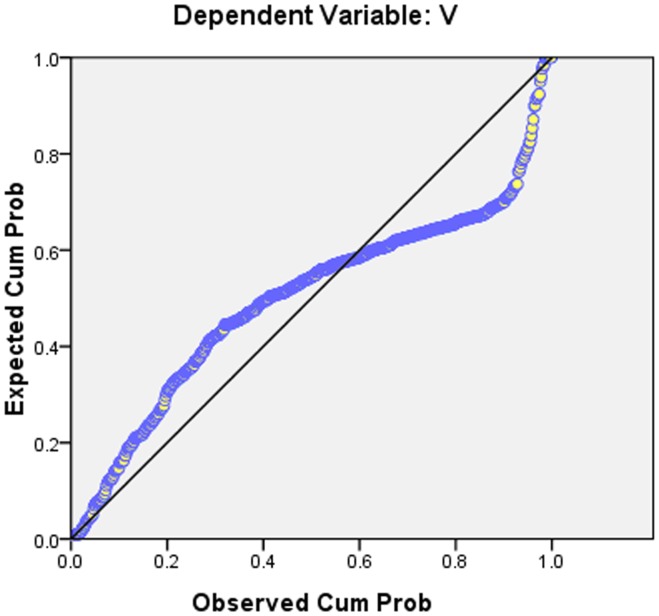
Cumulative probability distribution map.

It is known that the correlation coefficient of the multiple regression model of Beijing urban 3D green biomass was high(over 0.89) which correlated well with the 3D green biomass, remote sensing factors and GIS factors, and F values also showed that the significant differences existed in the model. The histogram of regression standardized residual in Figure 7 illustrated that it was an ideal bell-shaped normal distribution, and a better diagonal distribution was illustrated in the cumulative probability distribution (Figure 7 and Figure 8).

### Accuracy analysis

We performed 241 samples to identify whether the actual measured values and estimated values significant differences existed among the treatments, and when we did, we used the (3D Green Biomass)TGB test to determine which specific combinations of values differed significantly (Table 6)

**Table 6:** Testing Results of Precision Ratio of TGB Based-on SPOT5 Image.

	Confidence Level	
	a = 0.05	a = 0.01
Sample Number(n)	241	
Total of Actual Measured Values 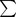	33687.621	
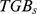 Average 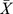	140.127	
Total of Estimated Values 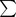	34788.741	
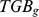 Average 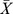	148.013	
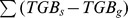	−1874.453	
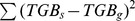	3513600.322	
Standard Deviation	122.804	
Standard Error 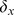	8.126	
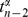	1.989	2.620
Absolute Error 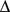	15.873	20.958
Relative Error E/%	10.695	14.204
Precision C/%	89.302	85.796

1. The residual standard deviation is acquired by formula 
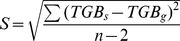
, where S is residual standard deviation, 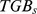 is the actual measured value of 3D green biomass, 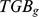 is the estimated value of 3D green biomass and *n* is the number of sampling plots for accuracy test.
2. The Standard error is acquired by formula 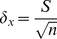 , where 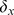 is Standard error.
3. The absolute error limit of 95% and 99% is calculated by formula 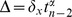 , where 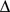 is the absolute error limit which acquired by *t* value distribution table difference.
4. The relative error limit of 95% and 99% is calculated by formula 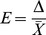 , where *E* is the relative error limit, and 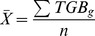 is the mean value of TGB.
5. Precision C is calculated by formula 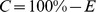.

The monitoring data in Table 6 demonstrated that the precision of the 3D green biomass of the sample based on the SPOT5 was over 85%, indicating that it could fully meet the requirements.

In all, based on the remote sensing image gray values extracted from the model by means of ArcGIS 9.3 and all statistical data calculated on the remote sensing retrieval model of the 3D green biomass, the green biomass in each region of Beijing could be determined. The results shows that the 3D green biomass of Beijing urban forest was 399.1295 million m^3^, of which coniferous was 28.7871 million m^3^ and broad-leaf was 370.3424 million m^3^.

## Discussion

As the above statistical data demonstrates, the case study described in this paper confirms that this is possible. Compared with the traditional 2D green indices in forest area, 3D green biomass represents an improvement over the conventional method because 3D index demonstrates that it can both accurately reflect the volume of the vegetation in the region, and scientifically assess the ecological environment of the city, while providing an important basis for urban planning and forest sciences development. The new approach of 3D green biomass illustrates that it is performed more accurately, efficiently, easily, and rapidly than the conventional, because the 3D green biomass not only involves the processing of remote sensing image including identification and classification, but also includes the investigation of forest vegetation on the ground, especially where the same species make great differences in different climatic zones.

**Figure 9:**
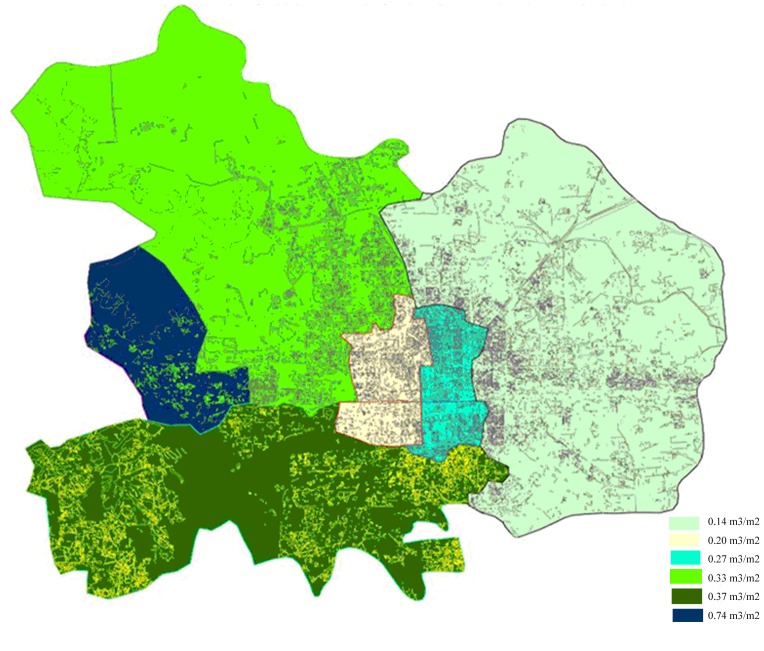
Grade distribution of 3D green biomass per unit area in Beijing.

The total 3D green biomass of Beijing urban areas can be acquired by the model and its grade distribution of biomass per unit can be also computerized in ArcGIS 9.3 shown in figure 9 [30], [31].

Beijing is an important capital urban district of China. In order to fully implement the strategy of “Humanistic Beijing, Scientific Beijing, Green Beijing” and promote the development of urban eco-environment, the study can provide some materials for references in urban green lands.

The crown form is one of non-negligible factors in the calculation of biomass with the help of 3D laser scanner, for example, the shape of the crown will shake in the wind when the scanned point cloud may not fully reflect the true state of the involved crown volume. Therefore, the wood should be scanned at rest to ensure the accuracy of the volume. Meanwhile, only the biomass from the upper half of crowns were involved while the under part, shrubs and herbs were not to be considered. In the future we will focus on the relative research in the field.

The green biomass of 3D in Beijing was estimated by the interpretation and classification of remote sensing data and modeling. In this paper, the urban vegetation was extracted by artificial visual interpretation and computer-aided, or strictly speaking it was still semi-automated and time and labor consuming. The extraction of vegetation is still the hot spot researchers interested in. The resolution in SPOT5 remote sensing image data was not high so that the crown width was greater than 2.5 m like a pixel. However, the gray on the corresponding band can produce the deviation if it is less than a pixel. In all, the biomass acquired in the study work as the exploratory research and reference and more remote sensing images with higher resolution will be used later to study.
